# Influence and interaction of resting state functional magnetic resonance and *tryptophan hydroxylase-2* methylation on short-term antidepressant drug response

**DOI:** 10.1186/s12888-022-03860-z

**Published:** 2022-03-25

**Authors:** Tingting Tan, Zhi Xu, Chenjie Gao, Tian Shen, Lei Li, Zimu Chen, Lei Chen, Min Xu, Bingwei Chen, Jiacheng Liu, Zhijun Zhang, Yonggui Yuan

**Affiliations:** 1 Department of Psychosomatics and Psychiatry, School of Medicine, Zhongda Hospital, Southeast University, Nanjing, 210009 People’s Republic of China; 2 Key Laboratory of Developmental Genes and Human Diseases, Ministry of Education, School of Medicine, Southeast University, Nanjing, 210009 People’s Republic of China; 3 Department of Psychiatric Rehabilitation, Wuxi Mental Health Center, Nanjing Medical University, WuXi, 214123 People’s Republic of China; 4 School of Medicine, Southeast University, Nanjing, 210009 People’s Republic of China; 5 Department of Psychology and Psychiatry, School of Medicine, Jinling Hospital, Nanjing University, Nanjing, 210018 People’s Republic of China; 6 Department of Anatomy, Medical School, Southeast University, Nanjing, 210009 People’s Republic of China; 7 Department of Epidemiology and Biostatistics, School of Public Health, Southeast University, Nanjing, 210009 People’s Republic of China; 8 Department of Nuclear Medicine, School of Medicine, Zhongda Hospital, Southeast University, Nanjing, 210009 People’s Republic of China; 9 Department of Neurology, School of Medicine, Zhongda Hospital, Southeast University, Nanjing, 210009 People’s Republic of China

**Keywords:** Major Depressive Disorder, Antidepressants, DNA methylation, *TPH2*, Resting-state functional MRI

## Abstract

**Background:**

Most antidepressants have been developed on the basis of the monoamine deficiency hypothesis of depression, in which neuronal serotonin (5-HT) plays a key role. 5-HT biosynthesis is regulated by the rate-limiting enzyme tryptophan hydroxylase-2 *(TPH2*). *TPH2* methylation is correlated with antidepressant effects. Resting-state functional MRI (rs-fMRI) is applied for detecting abnormal brain functional activity in patients with different antidepressant effects. We will investigate the effect of the interaction between rs-fMRI and *TPH2* DNA methylation on the early antidepressant effects.

**Methods:**

A total of 300 patients with major depressive disorder (MDD) and 100 healthy controls (HCs) were enrolled, of which 60 patients with MDD were subjected to rs-fMRI. Antidepressant responses was assessed by a 50% reduction in 17-item Hamilton Rating Scale for Depression (HAMD-17) scores at baseline and after two weeks of medication. The RESTPlus software in MATLAB was used to analyze the rs-fMRI data. The amplitude of low-frequency fluctuation (ALFF), regional homogeneity (ReHo), fractional ALFF (fALFF), and functional connectivity (FC) were used, and the above results were used as regions of interest (ROIs) to extract the average value of brain ROIs regions in the RESTPlus software. Generalized linear model analysis was performed to analyze the association between abnormal activity found in rs-fMRI and the effect of *TPH2* DNA methylation on antidepressant responses.

**Results:**

Two hundred ninety-one patients with MDD and 100 HCs were included in the methylation statistical analysis, of which 57 patients were included in the further rs-fMRI analysis (3 patients were excluded due to excessive head movement). 57 patients were divided into the responder group (*n* = 36) and the non-responder group (*n* = 21). Rs-fMRI results showed that the ALFF of the left inferior frontal gyrus (IFG) was significantly different between the two groups. The results showed that *TPH2–1–43* methylation interacted with ALFF of left IFG to affect the antidepressant responses (*p* = 0.041, false discovery rate (FDR) corrected *p* = 0.149).

**Conclusions:**

Our study demonstrated that the differences in the ALFF of left IFG between the two groups and its association with *TPH2* methylation affect short-term antidepressant drug responses.

## Background

Major depressive disorder(MDD)is a serious mental disorder with high morbidity, disability, recurrence rate, and suicide rate [1]. According to the World Health Organization (WHO), 322 million people suffer from MDD worldwide, which is about 4.4% of the global population [2, 3]. Depression is considered a global health issue and a major contributor to the global burden of disease (GBD). MDD is expected to top the GBD list by 2030 [4].

Many treatments for patients with MDD have been developed in recent years, such as physical and psychological therapies (including repetitive transcranial magnetic stimulation [rTMS] and electroconvulsive therapy [ECT]) and cognitive-behavioral therapy (CBT) [5–7]. However, use of antidepressants is the gold standard for treating depression [8]. Globally, two-thirds of patients with MDD fail to experience adequate response or remission after antidepressant treatment and develop treatment-resistant depression (TRD) [9, 10].

To improve the effectiveness of antidepressants, researchers have been identifying biomarkers that can predict antidepressant responses so as to develop personalized and optimal clinical therapies. Several biomarkers have been developed as predictors of antidepressant treatment responses, such as brain imaging [11, 12], genetic information [13–15] and levels of inflammatory molecules in the peripheral blood [16, 17].

Most antidepressants were developed on the basis of the monoamine deficiency hypothesis of depression [18, 19]. These drugs increase serotonin (5-HT) levels in the synaptic cleft through prohibitive binding to 5-HT transporters (5-HTTs), consequently enhancing 5-HT neurotransmission and causing an antidepressant effect [20, 21]. Thus, 5-HT is critical for 5-HT synthesis and antidepressant effects. Tryptophan hydroxylase-2 (*TPH2*) is a rate-limiting enzyme for 5-HT synthesis in the central nervous system [22, 23]. Previous studies have reported that *TPH2* genetic variants play an important role in antidepressant responses, and the *TPH2* methylation status is related to antidepressant effects [24–26].

In addition to epigenetic studies on the effects of antidepressants, previous studies have shown that magnetic resonance imaging (MRI), particularly functional MRI (fMRI), is highly useful in studying antidepressant responses as this technique helps in analyzing changes in the brain structure and functional activity [27, 28]. MRI monitors functional brain activities and can directly measure how different brain regions are involved in various brain activities [29]. As the fMRI is capable of showing very early synaptic changes after antidepressant exposure, it can be used to observe antidepressant responses [30, 31].

Resting-state fMRI (rs-fMRI), a type of fMRI, has been used to evaluate spatial functional correlations within neural networks in the resting state [32]. Recently, rs-fMRI has been widely used to investigate abnormal brain function in antidepressant responders and non-responders [33]. Aizenstein et al. found that compared with non-responders, responders showed increased functional connectivity (FC) of the emotion-related region of the brain [34]. Emam1 et al. reported that compared to the responder group, the non-responder group showed significantly less amplitude of frequency fluctuations (ALFF) in the dorsomedial prefrontal cortex (dmPFC) [35]. Thus, rs-fMRI is an important technique that can be used to explore antidepressant effects.

Wheater et al. reported that a combination of DNA methylation status and fMRI data could better explain the changes in disease phenotypes [36]. Until now, the few studies have used this combination approach to study depression [37–39]. However, none of these studies explored the effects of antidepressants using this combination approach. These studies have the following limitations: 1. they focused on FC in particular brain regions without considering abnormal functional activities in other brain regions; 2. the correlation between DNA methylation status and rs-fMRI has been studied, but the interaction between them has not been studied has not been lucidated; 3. these studies only focused on two common genes (*FKBP5* and *SLC6A4*) and not *TPH2*.

Therefore, in this study, we hypothesized that the functional activities in the brain regions between responders and non-responders to antidepressant medication were different, and the relationship between these activities and *TPH2* methylation could affect short-term antidepressant responses. Hence, we analyzed the differences in the rs-fMRI data between the responder and non-responder groups, explored the interaction between rs-fMRI data and *TPH2* methylation status, and determined the effect of this relationship on antidepressant effects.

## Methods

### Participants and clinical evaluation

In this study, we enrolled 300 patients with MDD at the Zhongda Hospital and 100 healthy controls (HCs). All the patients with MDD met the criteria mentioned in the Diagnostic and Statistical Manual of Mental Disorders, Fourth Edition (DSM-IV) [40]. Blood samples were collected from all the participants before they were administered antidepressants. In addition, one-fifth of the MDD patients (60) underwent fMRI scanning before starting antidepressant treatment.

All the recruited participants belonged to the Han Chinese ancestry. The criteria for selecting patients to be included in the MDD group were as follows: (1) Age between 18 and 65 years; (2) duration of depressive disorder persisting for more than two weeks; (3) 17-item Hamilton Rating Scale for Depression (HAMD-17) scores of ≥ 17 at baseline; (4) no history of any disease that could affect rs-fMRI results, such as cardiovascular, liver, and kidney diseases; (5) newly diagnosed or recently relapsed patients were treatment-free for over two weeks. The patients with depression were diagnosed by two senior psychiatrists independently and confirmed by a third psychiatrist blinded to the previous evaluation.

The exclusion criteria of this study were as follows: (1) Participants with a history of brain organic mental disorder, endocrine and primary organic disorder, or other medical conditions that could disrupt mental assessments; (2) a history of substance abuse (drugs, caffeine, nicotine, alcohol, or other), head injury, or disturbances of consciousness; (3) patients who had received ECT 6 months prior to the study; (4) pregnant and lactating women. (5) a history of manic episodes within the preceding 12 months.

The study was performed in accordance with the Declaration of Helsinki and approved by the Zhongda Hospital ethics committee (2016ZDSYLL100-P01). Informed consent was obtained from all the participants.

### Antidepressant treatment and its outcome evaluation

All the patients received antidepressants for more than two weeks without additional psychological intervention and followed up for one year to monitor any changes in the diagnosis. The psychiatrists prescribed the most appropriate single antidepressant to the MDD patients (selective serotonin reuptake inhibitors (SSRIs): *n* = 177 and non-SSRIs: *n* = 114, which included serotonin and norepinephrine reuptake inhibitors (SNRI): *n* = 94, noradrenergic and selective serotonergic antidepressants (NaSSAs): *n* = 13, and serotonin antagonists and reuptake inhibitors (SARI): *n* = 7) prohibiting the use of mood stabilizers and antipsychotics or other antidepressants. However, in a few clinical situations, low doses of benzodiazepines were allowed. The patients received the defined dosage of the antidepressants throughout the study period. A meeting was convened for standardizing the assessment and treatment protocols used by the participating psychiatrists before study commencement. Patients received no extra psychological intervention except supportive medical interviews. The HAMD-17 was used to assess the severity of depressive symptoms after two weeks from baseline [41]. The treatment response was defined as ≥ 50% reduction in the baseline HAMD-17 scores after treatment according to World Federation of Societies of Biological Psychiatry guidelines (WFSBP) [42].

### DNA methylation analysis

Blood samples were collected from the participants at 8:00 a.m. after fasting for at least 8 h before receiving antidepressant treatment. Venous blood samples were collected from the patients and control group for methylation analysis in 5 mL EDTA vacutainer tube and stored at − 80 °C for later use. The amount of methylated DNA was analyzed by MethylTarget® (Genesky Biotechnologies Inc., Shanghai, China) by NGS-based, multiple-targeted CpG methylation analysis. Polymerase chain reaction (PCR) primers for the target regions were designed using the Methylation Primer software. The samples were treated with sodium bisulfite using the EZDNA Methylation Kit (Zymo Research, Irvine, CA, USA) and according to the manufacturer’s instructions. A PCR mixture was prepared for each reaction (Takara, Tokyo, Japan) and consisted of Mg^2+^, dNTP, each primer, HotStaraq polymerase (Takara, Tokyo, Japan), appropriate buffer, and the template DNA. PCR amplicons (170–270 bp) were purified using the QIAquick Gel Extraction Kit (QIAGEN). The products were sequenced on an Illumina HiSeq platform using a pair-end 150 bp mode by following the manufacturer’s protocol. For all samples of the target regions, the average sequencing depth reached 600x; for over 80% of the samples, the depth was more than 400X, and for 90% of the samples, the depth was more than 10X.

Based on our previous studies [43, 44], only 11 single nucleotide polymorphisms (SNPs), meeting the standards for the methylation status of the sequence, were detected. Primers were designed to cover the upstream 100 bp and downstream 100 bp of 11 *TPH2* SNP sites, as well as the GC sequence content from cytosine-phosphate-guanine (CpG) sites > 20% after methylation. Methylation levels at 38 CpG sites of *TPH2* were determined along with the ratio of methylated cytosines over total cytosines. These values were tested and exported to Excel for statistical analysis.

### MRI analysis

MRI data were obtained using a 3.0-Tesla Siemens Trio MRI scanner using a 12-channel head coil. At baseline, the MRI scans were obtained at Zhongda Hospital of Southeast University. Each participant lay down supine with the head stabilized with cushions to minimize head movement. Additionally, the participants were provided with earplugs to lower the noise caused by the MRI scanner. High-resolution 3-dimensional T1-weighted images were obtained using a magnetization-prepared rapid gradient echo sequence: repetition time (TR) = 1900 ms; echo time (TE) = 2.48 ms; flip angle (FA) = 9°; acquisition matrix = 256 × 256; field of view (FOV) = 250 × 250 mm^2^; slice thickness = 1.0 mm, gap = 0; 176 sagittal slices; duration = 4 min 18 s. The rs-fMRI was used with the following sequence parameters: slices = 36; TR = 2000 ms; TE = 25 ms; FA = 90; acquisition matrix = 64 × 64; FOV = 240 mm × 240 mm; thickness = 3.0 mm; gap = 0 mm and 3.75 mm × 3.75 mm in-plane resolution parallel to the anterior commissure-posterior line. The scan time for one measurement lasted for about 8 min. During the fMRI scans, all participants were asked to be awake, to keep their eyes closed, relax, and refrain from any specific thinking [45].

The rs-fMRI data were preprocessed using the software MATLAB 2014a and Rs-fMRI Data Analysis Toolkit (REST; http://restfmri.net) [46]. Preprocessing included the following steps: (1) The first ten time points were discounted to ensure stable-state longitudinal magnetization and to allow subjects acclimate to the scanning environment; (2) functional images were corrected for differences in image acquisition time between slices; (3) all the imaging data were realigned for head movement correction; (4) the participant’s anatomical images were segmented, and the deformation field maps were applied to the functional images to normalize them into the standard Montreal Neurological Institute (MNI) space, with a resampled voxel of size 3 × 3 × 3 mm^3^ [47]; (5) the functional volumes were spatially smoothed by means of an isotropic Gaussian kernel of 6 mm full-width at half-maximum. (6) the linear trend of the time course was removed; (7) the regression of nuisance covariates including Friston 24 head-motion parameters, cerebrospinal fluid signals, global mean signals and white matter signals from the fMRI data were performed [48]; (8) Data were band-pass filtered to retain frequencies between 0.01 and 0.08 Hz [49]; (9) For ReHo, the calculation was carried out first and then spatial smoothing was performed [52].

The rs-fMRI data were analyzed using ALFF, regional homogeneity (ReHo), and fractional ALFF (fALFF) to measure the local spontaneous activity of individual regions or voxels, and seed-based FC was used to measure functional relationship with the seed region in the whole brain. For ALFF, the fast Fourier transform was used to transform the time series for each voxel to the frequency domain to obtain the power spectrum, and the square root of the power spectrum was calculated and averaged across 0.01–0.08 Hz [50]. FALFF data was computed with the ratio of the power spectrum of a given frequency range to the entire frequency range, which significantly improved the sensitivity and specificity in detecting the regional spontaneous brain activity [51]. ReHo was used for assessing the local temporal synchronizations by calculating Kendall’s coefficient of concordance between the time series of a given voxel and its nearest 26 neighbors [52].

Based on the ALFF, fALFF, and ReHo results, we performed seed-based resting-state FC (rs-FC) analysis to identify the changes in the brain networks that were centered in the regions showing abnormal local spontaneous activity in the responder and non-responder groups [53]. The above results were used as regions of interest (ROIs) for further research.

The Exsignal method in the RESTPlus software in MATLAB 2014a was used to extract the abnormal activity values of the brain regions with differences in the above four rs-fMRI processing methods, which were then converted into Excel for statistical analysis.

### Statistical analysis

All statistical analyses were performed using the IBM SPSS Statistical software package version 25 (IBM, Chicago, IL, USA). Means and standard deviations were considered continuous variables. *P*-values < 0.05 indicated statistical significance. T-test and Mann–Whitney U tests were used to identify differences in demographic characteristics between groups. For determining the CpG-site methylation status between groups, since 12 CpG sites were distributed normally (Shapiro–Wilk test, all *p*-values > 0.05), the remaining 26 CpG sites (Shapiro–Wilk test, all *p*-values < 0.05) were compared with t-tests and Mann–Whitney U tests separately. The false discovery rate (FDR) was applied to correct a large number of individually tested CpG sites. The differences in the above rs-fMRI parameters between the responder and non-responder groups were assessed using the two-sample test (*p* < 0.05). All regression analyses mentioned previously were controlled for age, gender, and education. Gaussian random field (GRF) corrections were used for all multiple comparison corrections (a voxel level of *p* < 0.005 and a cluster-level of *p* < 0.05). Based on previous results of our research team, the *TPH2* gene methylation sites associated with depression and antidepressant effects (*TPH2–7–184*, *TPH2–10–60*, *TPH2–1–43*, *TPH2–9–178*, *TPH2–5–203*, *TPH2–7–142* and *TPH2-8–237*) were included in the statistical analyses [26]. Median values were used as a cut-off point to divide the above sites into hypomethylation and hypermethylation groups [26]. Participants were divided into two categories according to the cut-off value of an average of mean ALFF (mALFF) ROI, which mALFF ROI high group and mALFF ROI low group [54]. The generalized linear model was used to test the effects of the mALFF ROI, *TPH2* gene methylation and mALFF ROI-*TPH2* gene methylation interaction on the antidepressant effects. The classification variables mALFF ROI and *TPH2* gene methylation were taken as independent variables to construct a model of the relationship between mALFF ROI and *TPH2* gene methylation as well as their interaction with antidepressant effects, while age and gender were taken as covariables. R programming language (Version 1.2.1335, Vienna, Austria) was used to perform the false discovery rate (FDR) test with a statistical significance of *p* < 0.05.

## Results

All the recruited patients with MDD had completed the two-week antidepressant treatments. During the 2-week follow-up period, one participant did not appear for the blood testing, one patient with depression withdrew from the study, and seven were retrospectively excluded at the one-year follow-up after diagnostic modification. 291 patients with MDD and 100 healthy controls were included in the methylation statistical analysis. Demographic and clinical characteristics of the 291 MDD patients and the 100 controls are detailed in Shen's article [26]. A total of 57 patients were included in the further analysis (3 patients were excluded due to excessive head movement). According to the reduction ratio of HAMD-17, 57 patients with MDD were divided into two groups: 37 patients who responded to antidepressant treatments (responders) and 21 patients who failed to respond to the antidepressant treatments (non-responders). There were no statistical significances in age (*p* = 0.552) and gender (*p* = 0.333) in these 57 patients with MDD (Table 1).

**Table 1:** Demographic characteristics between responders and non-responders

	Responders (*N* = 36)	Non-responders (*N* = 21)	t	*p* value
Gender Male/female(%)	27.8%/72.2%	42.9%/57.1%	0.053	0.333
Age (mean ± SD)	42.970 ± 2.220	45.620 ± 2.910	0.027	0.552
TPH2-1–43 (mean ± SD)	0.560 ± 0.080	0.480 ± 0.110	-0.055	0.053
ALFF ROI (mean ± SD)	0.580 ± 0.080	0.100 ± 0.060	0.054	0.060

*ALFF* Amplitude of low-frequency fluctuation, *ROI* Regions of interests, *SD* Standard deviation, *TPH2 Tryptophan hydroxylase*2

**Fig.1:**
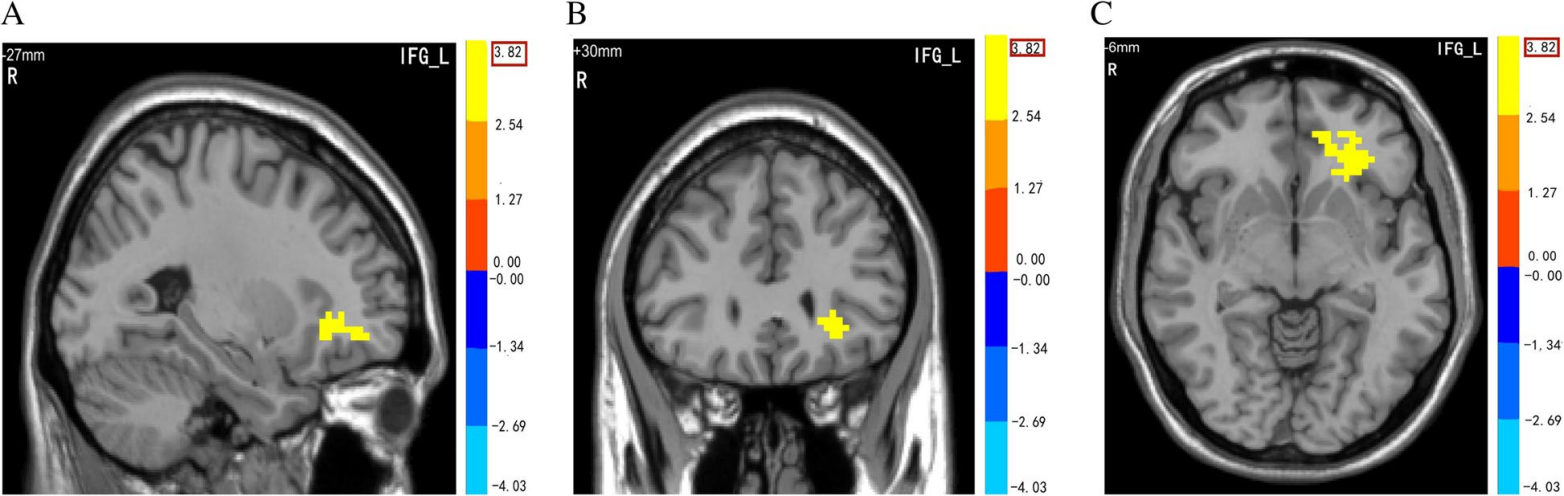
Different ALFF in Left IFG between responders and non-responders. ALFF–Amplitude of low-frequency fluctuation.; IFG- Inferior frontal gyrus; L-Left; R-Right

Our rs-fMRI results showed differences in the ALFF of the left inferior frontal gyrus (IFG) between the responders and non-responders (Fig. 1 A, Fig. 1 B, Fig. 1 C, Table 2), which coordinate is (-27 30 -6). There was no difference in the brain regions between the two groups according to ReHo and fALFF analyses. Based on the regional differences between groups found in the mALFF analysis, the FC analyses of the rs-fMRI data revealed that no difference in the brain regions was observed between the two groups.

**Table 2:** Brain areas with significantly different ALFF values between responders and non-responders

Brain areas	MNI coordinates	Number of voxels	Peak t-value
	X Y Z		
L- IFG	-27 30 -6	84	3.8158

A *P*-value < 0.05 was significantly different for multiple comparisons using Gaussian random field theory. *ALFF* Amplitude of low-frequency fluctuation, *MNI* Montreal Neurological Institute, *L* Left, *IFG* Inferior frontal gyrus

**Fig.2:**
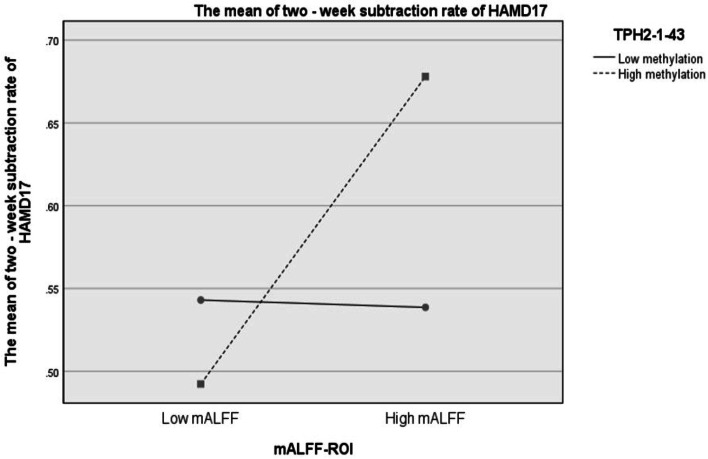
Effect of mALFF ROI and *TPH2-1–43* interaction on 2-week antidepressant responses. mALFF–mean Amplitude of low-frequency fluctuation; ROI-Regions of interest; HAMD17-17-item Hamilton Rating Scale for Depression; *TPH2-Tryptophan hydroxylase2*

The result showed that low left IFG ALFF significantly interacted with *TPH2-1–43* low methylation levels in affecting antidepressant responses(B value = 0.190, Wald Chi-Square = 4.168, *p* = 0.041, standard error(SE) = 0.931, 95% Wald Confidence Interval, Lower = 0.008, Upper = 0.373, FDR corrected *p* = 0.149)(Fig. 2, Table 3). MDD patients with *TPH2-1–43* low methylation level and low mALFF in the left IFG could have significantly better antidepressant responses. The results further suggested that antidepressant effects in MDD patients with low left IFG mALFF is significantly lower than that those with high mALFF (B value = -0.186, Wald Chi-Square = 8.103, *p* = 0.004, SE = 0.653, 95% Wald Confidence Interval, Lower = -0.314, Upper = -0.058, FDR corrected *p* = 0.149). Analysis showed no significant differences in the *TPH2-1–43* DNA methylation levels between the two groups (B value = -0.139, Wald Chi-Square = 3.758, *p* = 0.053, SE = 0.071, 95% Wald Confidence Interval, Lower = -0.280, Upper = -0.002,FDR corrected *p* = 0.149)(Table 3).

**Table 3:** Interaction effect of TPH2 methylation and ALFF ROI on HAMD 2-week reduction rate. *mALFF* mean Amplitude of low-frequency fluctuation, *ROI* Regions of interest, *p < 0.05, *TPH2 Tryptophan hydroxylase2*, *SE* standard error

	B value	Wald Chi-Square	SE	95% Wald Confidence Interval	*p* value
				Lower Upper	
*TPH2-1–43*					
Low Level	-0.139	3.758	0.071	-0.280 -0.002	0.053
High Level	-	-	-	- -	-
mALFF ROI					
Low Level	-0.186	8.103	0.653	- 0.314 -0.058	0 .004*
High Level	-	-	-	- -	-
*TPH2-1–43* * mALFF ROI					
Low Level * Low Level	0.190	4.168	0.931	0.008 0.373	0.041*
Low Level * High Level	-	-	-	- -	-
High Level * Low Level	-	-	-	- -	-
High Level * High Level	-	-	-	- -	-
Age	0.001	0.353	0.002	-0.163 0.055	0.552
Gender	-0.054	0.938	0.056	0.008 0.373	0.333

## Discussion

Differences in ALFF results of the left IFG between the responders and non-responders were observed in our study. Yang et al. reported that the therapeutic effects of antidepressants could be achieved by changing the FC in the hypothalamic regions, mainly in the inferior frontal, cingulate gyrus thalamus, and cerebellum [55]. Ichikawa et al. reported that antidepressants affect the FC between the left dorsolateral prefrontal cortex (DLPFC)/ IFG and posterior cingulate cortex (PCC)/precuneus to produce different effects of antidepressant treatments [56]. Our findings are comparable to those of previous studies that have reported functional abnormalities in IFG. However, these findings suggest that FC of the IFG may influence the antidepressant effects.

The left IFG is a key region for language comprehension and production. It also plays an important role in language processing [57, 58]. However, previous studies have demonstrated that left IFG is also a site of convergence of cognitive and emotional information, involved in the affective aspects of language processing, semantics, and visual memory [59]. A previous study showed the left IFG as an important area for emotional processing and belongs to a top-down cognitive control network during emotion processing that is responsible for understanding and controlling emotions [60]. Previous studies demonstrated that MDD patients had increased ALFF in the frontal lobe after antidepressant treatments [61]. These results indicated that a return to normal frontal lobe activity is associated with improvement in antidepressant efficacy. So, we speculate that for the patients with the higher spontaneous brain activity in the left IFG, the brain area activity may be more easily affected by the top-down modulatory network, so as to restore the normal emotional processing.

Previous studies have documented that FC of the right IFG and the orbitofrontal cortex was related to antidepressant effects [62]. Additionally, other studies have reported the FC of bilateral IFG and inferior frontal gyrus was related to antidepressant responses [55, 56]. Our results are inconsistent with those of previous studies. It is possible that the sample size of our study was small, which may reduce the effect of FC in the left IFG on antidepressant effects. Further studies are required to explore the relationship between the FC in the left IFG and antidepressant responses.

Moreover, our study explained the interaction between ALFF in the left IFG and *TPH2* methylation affecting antidepressant effects. Although there are no studies investigating the effect of interaction with rs-fMRI and DNA methylation on antidepressant responses. So far, only a few studies had focused on the relationship between them and MDD. Julian Chiarella et al. reported that *FKBP5* methylation, which is associated with depression, was related to the FC between the left orbitofrontal cortex (OFC) and the frontal lobe-limbic cortex [37]. Another study reported a strong positive correlation between level of *SLC6A4* methylation, which is associated with depression, and FC in the amygdala in a healthy population [63]. Meanwhile, Elmira Ismaylovaer et al. had displayed that blood-derived SLC6A4 methylation was positively associated with right lateral parietal area (RLP)-frontal pole regional rs-FC [64]. Additionally, Hedi Foo et al. had analyzed and summarized the above research results, then proposed that epigenetic variation could affect the FC of brain regions which may cause disease resilience/susceptibility [65]. According to the above results, it is proved that FC of brain regions is closely correlated with DNA methylation. Therefore, we have speculated that the functional activities of local brain regions may be correlated with DNA methylation. However, there is no studies have explored the relationship between functional activities of local brain regions and DNA methylation status. Based on the assumption, our research has demonstrated that the interaction existed between DNA methylation and functional activities of local brain regions, and the interaction will eventually affect the efficacy of antidepressants. Thus, it is crucial to focus on the relation between DNA methylation and functional activities of local brain regions in future studies.

In this study, we found that the interaction between hypomethylation levels of *TPH2-1–43* and low ALFF level in the left IFG can lead to the better antidepressant responses. However, we also found that antidepressant responses could be reduced in MDD patients who had low spontaneous activity compared with high spontaneous activity. Moreover, previous research of our group demonstrated that the lower the methylation level of TPH2-1–43, the worse the antidepressant effect [26]. Hence, our results suggested a mutually exclusive interaction between the methylation level of *TPH2-1–43* and the spontaneous activity of the left IFG. It may explain that the low methylation level of *TPH2-1–43* can antagonize the worse antidepressant efficacy caused by low spontaneous activity in the left IFG. The susceptibility of MDD is influenced by the interactions between genomic variants and environmental factors [66]. Particularly, DNA methylation stands for the main mediators of the impact of the environment in increasing the vulnerability risk to develop MDD [67]. DNA methylation could play an important role in brain development and function [68]. Hence, it may eventually lead to influence on the antidepressant effects. In addition, our results also suggested that researchers wanting to identify the biomarkers of antidepressant effects could take into account the interaction between DNA methylation and rs-fMRI. Hence, it may eventually lead to influence on the antidepressant effects. In addition, our results also suggested that researchers wanting to identify the biomarkers of antidepressant effects could take into account the interaction between DNA methylation and rs-fMRI. Based on our results, we speculated that *TPH2* methylation may cause abnormal functional activity in the brain regions and ultimately lead to differences in antidepressant effects (such as ALFF). Unfortunately, we needed to point out that this result did not pass the FDR threshold at *p* = 0.041. The reasons may be that lack of strong interaction between DNA methylation status and brain function and the small sample size.

Limitations of the present study are worth noting. The sample sizes in this study were relatively small; Moreover, the *TPH2* methylation status-related information and rs-fMRI data were obtained only at baseline and not after 2-week antidepressant treatment. Therefore, future studies with larger sample sizes are necessary to detect gene DNA methylation and rs-fMRI to confirm our findings. Finally, the fact that our study failed to show relationship between types and doses of antidepressant drugs and the identification of the difference regions. Future studies need to consider the types and dosages of antidepressant drugs to reduce the effect of the heterogeneity and doses difference of drugs on recognition of abnormal brain regions.

## Conclusions

Our study suggests that the differences in the ALFF in the left IFG and its association with *TPH2* methylation status affect short-term antidepressant drug response. Further research is needed to explore how the relationship between DNA methylation and functional changes in the brain regions affect antidepressant responses. It may help in better guiding treatment decisions and hastening clinical response.

## Data Availability

The datasets used and/or analysed during the current study are available from the corresponding author on reasonable request.

## Abbreviations

ALFF: Amplitude of low-frequency fluctuation
CBT: Cognitive-behavioral therapy
CpG: Cytosine-phosphate-guanine
DLPFC: Dorsolateral prefrontal cortex
DmPFC: Dorsomedial prefrontal cortex
DSM-IV: Diagnostic and Statistical Manual of Mental Disorders, Fourth Edition
ECT: Electroconvulsive therapy
FA: Flip angle
FALFF: Fractional ALFF
FC: Functional connectivity
FDR: False discovery rate
FMRI: Functional MRI
FOV: Field of view
GBD: Global burden of disease
GRF: Gaussian random field
HCs: Healthy controls
HAMD-17: 17-Item Hamilton Rating Scale for Depression
IFG: Inferior frontal gyrus
L: Left
mALFF: Mean ALFF
MDD: Major depressive disorder
MNI: Montreal Neurological Institute
MRI: Magnetic resonance imaging
NaSSAs: Noradrenergic and selective serotonergic antidepressants
OFC: Orbitofrontal cortex
PCC: Posterior cingulate cortex
PCR: Polymerase chain reaction
Qiagen: QIAquick Gel extraction kit
R: Right
ReHo: Regional homogeneity
RLP: Right lateral parietal
ROIs: Regions of interest
Rs-FC: Resting-state FC
Rs-fMRI: Resting-state functional MRI
RTMS: Repetitive transcranial magnetic stimulation
SARI: Serotonin antagonists and reuptake inhibitors
SD: Standard deviation
SE: Standard error
SNRI: Serotonin and norepinephrine reuptake inhibitors
SNPs: Single nucleotide polymorphisms
SSRIs: Selective serotonin reuptake inhibitors
TE: Echo time
*TPH2*: Tryptophan hydroxylase-2
TR: Repetition time
TRD: Treatment-resistant depression
WFSBP: World Federation of Societies of Biological Psychiatry guidelines
WHO: World Health Organization
5-HT: Serotonin
5-HTTs: 5-HT transporters
